# Entropy Production in Reaction–Diffusion Systems Confined in Narrow Channels

**DOI:** 10.3390/e26060463

**Published:** 2024-05-29

**Authors:** Guillermo Chacón-Acosta, Mayra Núñez-López

**Affiliations:** 1 Applied Mathematics and Systems Department, Universidad Autónoma Metropolitana-Cuajimalpa, Vasco de Quiroga 4871, Mexico City 05348, Mexico; 2 Department of Mathematics, Instituto Tecnológico Autónomo de México, Río Hondo 1, Col. Progreso Tizapán, Mexico City 01080, Mexico; mayra.nunez@itam.mx

**Keywords:** diffusion in confinement, narrow channels, reaction–diffusion systems, pattern formation, entropy production, Gray–Scott model

## Abstract

This work analyzes the effect of wall geometry when a reaction–diffusion system is confined to a narrow channel. In particular, we study the entropy production density in the reversible Gray–Scott system. Using an effective diffusion equation that considers modifications by the channel characteristics, we find that the entropy density changes its value but not its qualitative behavior, which helps explore the structure-formation space.

## 1. Introduction

Many chemical and biological systems exhibit ordered structures that emerge spontaneously and remain stable over time. These structures arise and persist due to processes involving mass transport and chemical reactions under far-from-equilibrium conditions. For instance, Turing’s diffusion-driven instability mechanism describes how small fluctuations in reaction–diffusion systems can be amplified, forming spatio-temporal patterns [1,2]. These patterns arise from the interaction between chemical species, one considered inhibitory and the other activating. Diffusion induces the system’s uniform steady state, which is stable in the absence of diffusion, to become unstable, promoting the emergence of patterns. Transport originates in zones with higher inhibitor concentrations that block the activator, whereas low inhibitor concentrations do not, allowing the activator to grow [2]. This phenomenon is counterintuitive since diffusion is generally associated with a decrease in concentration gradients over time. The reaction–diffusion systems that have been used to elucidate pattern formation typically involve non-linear reactions. They often focus only on forward reactions, assuming their rates significantly surpass those of reverse reactions that have been used to elucidate these phenomena [3]. Consequently, for a complete understanding of these processes’ thermodynamics further study is needed. In classical thermodynamics, the evolution of systems within the linear regime is predictable. However, far-from-equilibrium systems may evolve into organized structures known as dissipative structures, as revealed by some analyses of irreversible thermodynamics [4,5,6].

Numerical studies have revealed some particular connections between entropy production and the emergence of patterns. Entropy production analysis in cycles of chemical oscillations or self-organizing biological systems has become possible in some reversible systems [7]. For instance, in chemical oscillations like the Belousov–Zhabotinsky reaction or the Oregonator model, entropy production averaged over a cycle may exceed or fall below the corresponding unstable equilibrium state, depending on the bifurcation parameter [8]. These processes are relevant, for instance, in self-healing surfaces involving pattern formation [9] or in enzymatic reactions of photosynthetic models that exhibit standard oscillatory and damped dynamics [10]. However, establishing thermodynamic rules for studying the evolution of non-equilibrium patterns remains challenging.

The Gray–Scott model is a powerful tool capable of replicating various Turing-like patterns. This model finds practical applications, accurately describing the autocatalytic reactions observed in chemical and enzymatic reactions in biochemistry and biology [11]. The original Gray–Scott model corresponds to two irreversible reactions for *U* and *V*, with *P* being an inert product [12]. This system models the chemical reaction U+2V→3V, which consumes *U* and produces *V*. Therefore, the amount of both substances must be controlled to maintain the reaction. This is done by adding at the feed rate *f* and removing at the kill rate *k*. The reduction and subsequent removal of *V* can also be described by another chemical reaction V→P. For this reaction, *P* is an inert product, meaning it does not react, and the parameter *k* controls the rate of the second reaction. The Gray–Scott system is defined by two equations that describe the behavior of two reacting substances:

(1) $$\begin{array}{ccc} \frac{\partial U}{\partial t} & = & D_{u}\nabla^{2}U-UV^{2}+\left(1-U\right)f, \end{array}$$

(2) $$\begin{array}{ccc} \frac{\partial V}{\partial t} & = & D_{v}\nabla^{2}V+UV^{2}-\left(f+k\right)V, \end{array}$$

where $D_{u}$ and $D_{v}$ are the diffusion coefficients for each chemical species. In order to remove *V* faster than the growth of *U*, the rate *k* is added to *f* and multiplied by *V*, since it is assumed that the disappearance of *V* also depends on its concentration.

The reversible Gray–Scott system has been proposed to study entropy production [3], featuring a cubic autocatalytic reaction coupled with a first-order reaction. Escher, Ross [13], and Yoshida [14] also previously studied it in the context of continuous-flow well-stirred tank reactors (CSTR), where far-from-equilibrium situations can also be achieved. Its phase diagram presents a stationary-stable equilibrium point, saddle-node, and Hopf bifurcations. Heterogeneous structures typically arise near these bifurcation curves [15,16].

Entropy production in self-replicating pulses, initiated by perturbing the equilibrium point, has been investigated [3,17]. While entropy production increases with the size of each pulse, its rate gradually decreases. Thus, the entropy production value can serve as a thermodynamic metric for evaluating pattern stability. Additionally, total entropy change, incorporating outward entropy flux using a relative chemical potential, has been examined within this model [18,19]. Specific trends in entropy production evolution have been observed during the formation of heterogeneous structures. In contrast to uniform steady states, during this process a compromise occurs between maximization and minimization of entropy production, reflecting the influence of several interacting mechanisms [20].

Incorporating a thermal component into the system has revealed transitions between different morphologies of Turing patterns regulated by temperature [21]. These transitions occur with lower entropy production compared to other parameters, such as kinetic constants and reactant fluxes [22]. Formalisms such as the Boltzmann equation [23] and extended irreversible thermodynamics [24] have also drawn further insights into entropy production and entropy flows in reaction–diffusion systems.

On the other hand, the study of transport processes and reactions within confined domains holds significant interest due to its wide range of applications, including carbon nanotubes [25], zeolites [26], biosensors [27], biological membranes [28], separation processes [29,30], and microfluidics, among others [31]. Confinement plays a crucial role in influencing the transport properties by restricting the system’s accessible configuration space [32,33]. The Fick–Jacobs approximation for diffusion in narrow channels simplifies this process by assuming a fast equilibration of the transverse degrees of freedom. It focuses on the longitudinal motion by projecting along the channel axis, resulting in an effective one-dimensional equation with a position-dependent diffusion coefficient [33]. Numerous studies have proposed different functions of this diffusion coefficient under diverse conditions, such as in symmetrical channels [34,35,36,37], asymmetrical channels [38,39,40], channels with curved midlines [41,42,43], or on curved surfaces [44,45,46]. Additionally, confinement effects on chemical reactions [47] and pattern formation in reaction–diffusion systems [48], including diffusive predator–prey models [49] and long-range effects [50], were also explored. Significantly, the geometry of the channel walls introduces modifications to the Turing instability conditions, the dispersion relation, and the range of unstable modes [48].

This work studies how confinement in a narrow channel changes the entropy production density in a reaction–diffusion system. By introducing entropic flow into the Fick–Jacobs approximation, as in [51], it is clear that wall geometry affects entropy production. In the following section, we review the definitions of entropy production for reaction–diffusion systems, focusing on the reversible Gray–Scott model. Subsequently, we explore the effects of geometry on a confined reaction–diffusion system within the local Fick–Jacobs approximation and, particularly, within the mean-field effective framework. We conclude by presenting the effective entropy production calculated with both approaches and the results concerning entropy production density by diffusive flux.

## 2. Entropy Production in 2D Reaction–Diffusion Systems

Entropy production comprises two distinct components in reaction–diffusion systems, as discussed in [3,5,6,7,8]. The first component arises from chemical reactions’ entropy produced per unit area (or length). The second component is the rate of entropy production due to diffusion. This total entropy production is calculated by integrating the entropy production density across the entire spatial domain:

(3) $$\sigma =\sigma_{R}+\sigma_{D}.$$

The contribution of chemical reactions is

(4) $$\sigma_{R}=\sum_{j}\left(\nu_{j}^{+}-\nu_{j}^{-}\right)\ln\left(\frac{\nu_{j}^{+}}{\nu_{j}^{-}}\right),$$

where $\nu_{j}^{\pm }$ are the forward and backward reaction rates of the *j*-th reaction. Far from equilibrium, backward rates can be considered small, and the system’s affinity tends to infinity, leading to linear entropy production. In this scenario, chemical oscillations and structure formation can occur [4]. The contribution to entropy production density due to the diffusion of an ideal solution is the product of the diffusive flux and the corresponding driving force:

(5) $$\sigma_{D}=-\sum_{k}J_{k}\cdot F_{k}=-\sum_{k}\left(-D_{k}\nabla P_{k}\right)\cdot \nabla \ln P_{k}=\sum_{k}\frac{D_{k}}{P_{k}}\left[{\left(\frac{\partial P_{k}}{\partial x}\right)}^{2}+{\left(\frac{\partial P_{k}}{\partial y}\right)}^{2}\right],$$

where $D_{k}$ and $P_{k}$ are the diffusivity and concentration of the *k*-th chemical species; the gas constant *R*, to be multiplied on the right-hand side, is chosen as 1 for simplicity of calculation [3,7]. Unlike previous studies [18,19], the contribution of entropy flow into and out of the system is not considered.

Irreversible chemical reaction systems are commonly used to model pattern formation, with the reverse reactions often omitted, as mentioned earlier. Accordingly, entropy production diverges for irreversible chemical reactions, as seen in Equation (4). This divergence prevents the calculation and use of entropy production in irreversible reactions [19]. Therefore, reversible chemical reactions, such as the reversible Gray–Scott model, are considered, to overcome this issue. It was first introduced by Mahara et al. in [3,17]. However, Escher, Ross [13], and Yoshida [14] had already considered a similar model for CSTR. The model consists of a cubic autocatalytic reaction U+2V↔3V coupled with a first-order reaction V↔P corresponding to the following system of equations:

(6) $$\begin{array}{ccc} \frac{\partial U}{\partial t} & = & D_{u}\nabla^{2}U-k_{1}UV^{2}+\left(1-U\right)f+k_{-1}V^{3}, \end{array}$$

(7) $$\begin{array}{ccc} \frac{\partial V}{\partial t} & = & D_{v}\nabla^{2}V+k_{1}UV^{2}-\left(f+k_{2}\right)V-k_{-1}V^{3}+k_{-2}P, \end{array}$$

(8) $$\begin{array}{ccc} \frac{\partial P}{\partial t} & = & D_{p}\nabla^{2}P+k_{2}V-\left(f+k_{-2}\right)P, \end{array}$$

where *U*, *V*, and *P* are the 2D concentrations of the involved chemical species, $k_{i}$ and $k_{-i}$ are the rate constants of the forward and backward reactions, respectively, with i=1,2, and *f* is the feeding rate constant. When backward reaction rates vanish, the original Gray–Scott model is recovered. The second reaction rate and the feeding rate describe the system by fixing the first reaction rate, the diffusion coefficients, and the backward rates. This system can produce many different patterns, such as spots and lines, and there are even indications of regions where different patterns can coexist [15]. The stability analysis of the system and the regions of the phase diagram $\left(k_{2},f\right)$ for which this system can form patterns in one and two dimensions are well-documented [15,17,20]. Studies of the phase diagram of the system indicate that it is monostable outside the saddle-node bifurcation curve but bi-stable between the saddle-node bifurcation curve and the Hopf bifurcation curve. Heterogeneous structures are often found precisely around these bifurcation curves [20].

For simplicity and to minimize the system parameters when solving the equations, the control parameters chosen were $k_{1}=1$ for the first direct reaction, $k_{-1}=k_{-2}=10^{-3}$ for the reverse reactions, and the diffusion coefficients $D_{u}=2\times 10^{-5}$, $D_{v}=10^{-5}$ and $D_{p}=10^{-6}$. The initial condition was taken as ${\left(U,V,P\right)|}_{t=0}=\left(0.50,0.25,0.00\right)$, as adopted by previous contributions [3,20]. The system size in 2D is 100×100 with 2500 time steps, and Neumann boundary conditions were used.

The entropy production density for this 2D system is the sum of these two terms:

(9) $$\begin{array}{ccc} \sigma_{R} & = & \left(k_{1}UV^{2}-k_{-1}V^{3}\right)\ln\left(\frac{k_{1}U}{k_{-1}V}\right)+\left(k_{2}V-k_{-2}P\right)\ln\left(\frac{k_{2}V}{k_{-2}P}\right), \end{array}$$

(10) $$\begin{array}{ccc} \sigma_{D} & = & \frac{D_{u}}{U}\left[{\left(\frac{\partial U}{\partial x}\right)}^{2}+{\left(\frac{\partial U}{\partial y}\right)}^{2}\right]+\frac{D_{v}}{V}\left[{\left(\frac{\partial V}{\partial x}\right)}^{2}+{\left(\frac{\partial V}{\partial y}\right)}^{2}\right]+\frac{D_{p}}{P}\left[{\left(\frac{\partial P}{\partial x}\right)}^{2}+{\left(\frac{\partial P}{\partial y}\right)}^{2}\right], \end{array}$$

whereas total entropy production is

(11) $$\tilde{\sigma }={\;\int \int \;}_{\Omega }dA\left(\sigma_{R}+\sigma_{D}\right).$$

**Figure 1:**
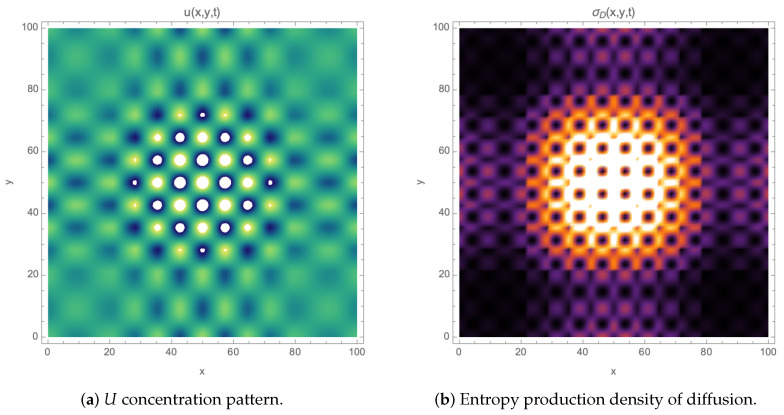
Patterns for the U(x,y,t) concentration and diffusive entropy production $\sigma_{D}\left(x,y,t\right)$ for the reversible Gray–Scott model Equations (6)–(8), both evaluated at t=2000. In (**a**), the color gradient ranges from bright yellow tones representing a higher concentration of U to dark blue tones indicating a lower concentration. In (**b**), bold tones reflect lower values of entropy density, while lighter tones indicate higher values.

In reference [17], it was observed that entropy production from reactive terms is approximately 20 times larger than that arising from the diffusion contribution, yet the latter remains important in affecting the dynamics of pattern formation. For this reason, we will only study the diffusive contribution, since the geometry of the boundaries will primarily influence this term. Figure 1 shows a typical two-dimensional pattern of this system and the corresponding diffusive contribution to the entropy density.

## 3. Pattern Formation in Reaction–Diffusion on Narrow Channels

When chemical species diffuse in a confined space, their transport is driven by stochastic dynamics and the available space for the motion, i.e., entropic effects. In the ideal case, it is possible to model the effect of periodic channel wrinkles on the diffusion of a particle as an overdamped Brownian motion in a one-dimensional periodic potential [52]:

(12) $$\gamma \dot{x}\left(t\right)=-V’\left(x\right)+F+\sqrt{2\gamma k_{B}T}\xi \left(t\right),$$

where γ is the friction coefficient, V(x) is the *L*-periodic potential, *F* is the static external force, $k_{B}$ is the Boltzmann constant, *T* is the temperature, and ξ is a Gaussian noise. In the free case, i.e., V=0, the Einstein relation $D_{0}=kT/\gamma$ is recovered. For the case F=0, the effective Lifson–Jackson coefficient for external potential is recovered [53]:

(13) $$D=\frac{D_{0}L^{2}}{\int_{0}^{L}dx\;\int_{0}^{L}dy\;e^{[V(x)-V(x-y)]/kT}}.$$

When considering non-interacting point particles diffusing in 2D, the process can be described by the corresponding Fokker–Planck equation associated to Equation (12), where the Neumann boundary conditions model the confinement.

It is well known that the system is well-approximated by 1D Brownian diffusion in the presence of entropic barriers [33]. In narrow channels characterized by a transverse length that is significantly smaller than the length of the channel axis, the diffusion equation can be projected to one effective dimension. In this case, the marginal concentrations of the *k*-species are

(14) $$\rho_{k}\left(x,t\right)=\int_{y_{1}\left(x\right)}^{y_{2}\left(x\right)}dy\;P_{k}\left(x,y,t\right),$$

where $y_{i}\left(x\right)$, i=1,2, are the functions that determine the geometry of the boundaries and allow us to introduce the so-called width function as $w\left(x\right)=y_{2}\left(x\right)-y_{1}\left(x\right)$. This projection assumes small amplitude boundary fluctuations $w^{\prime }\left(x\right)\ll 1$ and fast equilibration of the transverse degrees of freedom or uniform transverse concentrations. In this case, the 2D equation is replaced by the 1D Fick–Jacobs–Zwanzig operator:

(15) $$\frac{\partial }{\partial x}\left[D_{k}\left(x\right)w\left(x\right)\frac{\partial }{\partial x}\left(\frac{\rho_{k}\left(x,t\right)}{w(x)}\right)\right],$$

where $D_{k}\left(x\right)$ is the position-dependent diffusion coefficient of the species *k*, which usually depends on variations of the channel’s width function. According to [48], the reaction kinetic function changes correspondingly, as $wF(\rho_{k}/w)$. Also in [48], it was found that dispersion relations and the range of unstable modes will be affected by the geometry of the system and, therefore, the pattern formation. The major contribution was to explore the impact of channel geometry on reaction–diffusion systems and, consequently, on instability conditions for systems that are constrained by the effective one-dimensional projection technique.

However, the Fick–Jacobs equation is local and it is always desirable to have an equation defined in some particular domain. In [54], the authors present a theoretical link based on the macroscopic diffusion coefficient that is dependent on the porosity, tortuosity, and constriction parameters, by averaging the spatial-dependent diffusion coefficient obtained in the Fick–Jacobs (FJ) approximation. The authors compared the total fluxes predicted by the FJ reduction scheme and the effective medium theory with the numerical results arising from the two-dimensional diffusion equation, in order to show the use of the effective diffusion coefficient, given as follows:

(16) $$\frac{\partial {\hat{\rho }}_{k}}{\partial t}=\frac{D_{eff}^{k}W}{\langle w\rangle }\;\frac{\partial^{2}{\hat{\rho }}_{k}}{\partial x^{2}}$$

where

$$D_{eff}^{k}=D_{k}\frac{\phi \;\delta }{\tau },$$

In Equation (16), ${\hat{\rho }}_{k}$ is the averaged marginal concentration; $D_{eff}^{k}$ is the mean-effective diffusion coefficient, in terms of constriction factor δ, tortuosity τ, and porosity ϕ=〈w〉/W, where *W* is the approximate width of the rectangular domain containing the pore. This coefficient is analogous to the Lifson–Jackson averaged coefficient, applicable only to periodic channels [53]. By contrast, the coefficient in Equation (16) can be used for any pore, as long as its length is much greater than its width.

## 4. Effect of Channel Geometry on Entropy Production

### 4.1. Fick–Jacobs Approximation for Reaction–Diffusion Equations and Entropy Production

Accordingly, by constraining the dynamics of the reversible Gray–Scott model to a narrow channel domain, the system of equations becomes as follows:

(17) $$\begin{array}{ccc} \frac{\partial u}{\partial t} & = & \frac{\partial }{\partial x}\left[D_{u}\left(x\right)w\left(x\right)\frac{\partial }{\partial x}\left(\frac{u(x,t)}{w(x)}\right)\right]-\frac{k_{1}}{w^{2}}uv^{2}+\left(1-\frac{u}{w}\right)\frac{f}{w}+\frac{k_{-1}}{w^{2}}v^{3}, \end{array}$$

(18) $$\begin{array}{ccc} \frac{\partial v}{\partial t} & = & \frac{\partial }{\partial x}\left[D_{v}\left(x\right)w\left(x\right)\frac{\partial }{\partial x}\left(\frac{v(x,t)}{w(x)}\right)\right]+\frac{k_{1}}{w^{2}}uv^{2}-\left(f+k_{2}\right)v-\frac{k_{-1}}{w^{2}}v^{3}+k_{-2}p, \end{array}$$

(19) $$\begin{array}{ccc} \frac{\partial p}{\partial t} & = & \frac{\partial }{\partial x}\left[D_{p}\left(x\right)w\left(x\right)\frac{\partial }{\partial x}\left(\frac{p(x,t)}{w(x)}\right)\right]+k_{2}v-\left(f+k_{-2}\right)p, \end{array}$$

In this case, u,v,p are the Equation (14) marginal concentrations, noting also that some reaction rates are modified by the channel geometry through the width function.

The chemical contribution to the entropy production density at the channel is

(20) $$\sigma_{r}=\frac{1}{w^{2}}\left(k_{1}uv^{2}-k_{-1}v^{3}\right)\ln\left(\frac{k_{1}u}{k_{-1}v}\right)+\left(k_{2}v-k_{-2}p\right)\ln\left(\frac{k_{2}v}{k_{-2}p}\right).$$

whereas for the effective diffusive component, the Fick–Jacobs probability current and the corresponding driving force, respectively, are given as follows [51]:

(21) $$\begin{array}{ccc} J_{k} & = & -D_{k}\left(x\right)w\left(x\right)\frac{\partial }{\partial x}\left(\frac{\rho_{k}\left(x,t\right)}{w(x)}\right), \end{array}$$

(22) $$\begin{array}{ccc} F_{k} & = & \frac{\partial }{\partial x}\ln\left(\frac{\rho_{k}}{w}\right), \end{array}$$

and so the contribution to the entropy is $\sigma_{d}=-\sum_{k}J_{k}F_{k}$; thereby,

(23) $$\begin{array}{ccc} \sigma_{d} & = & \sum_{k}\frac{D_{k}\left(x\right)}{\rho_{k}\left(x,t\right)}{\left[w\frac{\partial }{\partial x}\left(\frac{\rho_{k}}{w}\right)\right]}^{2}=\sum_{k}\frac{D_{k}\left(x\right)}{\rho_{k}\left(x,t\right)}{\left(\frac{\partial \rho_{k}}{\partial x}-\frac{\rho_{k}}{w}\frac{\partial w}{\partial x}\right)}^{2}, \end{array}$$

(24) $$\begin{array}{ccc} & = & \frac{D_{u}\left(x\right)}{u}{\left[w\frac{\partial }{\partial x}\left(\frac{u}{w}\right)\right]}^{2}+\frac{D_{v}\left(x\right)}{v}{\left[w\frac{\partial }{\partial x}\left(\frac{v}{w}\right)\right]}^{2}+\frac{D_{p}\left(x\right)}{p}{\left[w\frac{\partial }{\partial x}\left(\frac{p}{w}\right)\right]}^{2}. \end{array}$$

Note that if the channel does not vary with the longitudinal coordinate we recover the standard 1D case. Instead, if $w^{\prime }\neq 0$, some terms dependent on the variations of the channel geometry arise, i.e., entropic forces modifying the entropy production density emerge.

### 4.2. Effective Diffusion Coefficient in Terms of Availability, Tortuosity, and Constriction

Using the approach of effective diffusion coefficients based on the channel’s geometric features, the problem is simplified to a one-dimensional system. The averaged concentration is defined as

$${\hat{\rho }}_{k}=\frac{1}{w}\int_{0}^{L}dy\;P_{k}.$$

From this perspective, the reaction–diffusion system becomes a 1D system with modified diffusion coefficients:

(25) $$\begin{array}{ccc} \frac{\partial \hat{u}}{\partial t} & = & \frac{D_{eff}^{u}W}{\langle w\rangle }\;\frac{\partial^{2}\hat{u}}{\partial x^{2}}-k_{1}\hat{u}{\hat{v}}^{2}+\left(1-\hat{u}\right)f+k_{-1}{\hat{v}}^{3}, \end{array}$$

(26) $$\begin{array}{ccc} \frac{\partial \hat{v}}{\partial t} & = & \frac{D_{eff}^{v}W}{\langle w\rangle }\;\frac{\partial^{2}\hat{v}}{\partial x^{2}}+k_{1}\hat{u}{\hat{v}}^{2}-\left(f+k_{2}\right)\hat{v}-k_{-1}{\hat{v}}^{3}+k_{-2}\hat{p}, \end{array}$$

(27) $$\begin{array}{ccc} \frac{\partial \hat{p}}{\partial t} & = & \frac{D_{eff}^{p}W}{\langle w\rangle }\;\frac{\partial^{2}\hat{p}}{\partial x^{2}}+k_{2}\hat{v}-\left(f+k_{-2}\right)\hat{p}, \end{array}$$

where $D_{eff}^{k}$ is the effective diffusion coefficient for each species, now dependent on geometric factors such as constriction, porosity, and tortuosity, which vary according to the specific shape of each channel.

The density of entropy production from reaction will be the same as in the one-dimensional case:

(28) $${\hat{\sigma }}_{r}=\langle w\rangle \left(k_{1}\hat{u}{\hat{v}}^{2}-k_{-1}{\hat{v}}^{3}\right)\ln\left(\frac{k_{1}\hat{u}}{k_{-1}\hat{v}}\right)+\langle w\rangle \left(k_{2}\hat{v}-k_{-2}\hat{p}\right)\ln\left(\frac{k_{2}\hat{v}}{k_{-2}\hat{p}}\right),$$

whereas the one corresponding to diffusion flux is as follows:

(29) $$J_{k}=-D_{eff}^{k}W\;\frac{\partial {\hat{\rho }}_{k}}{\partial x},\;F_{k}=\frac{\partial }{\partial x}\ln{\hat{\rho }}_{k},$$

such that

(30) $${\hat{\sigma }}_{d}=W\sum_{k}\frac{D_{eff}^{k}}{{\hat{\rho }}_{k}}{\left(\frac{\partial {\hat{\rho }}_{k}}{\partial x}\right)}^{2}.$$

It is worth noting that all these equations apply to the averaged concentration. Therefore, to compare with the one-dimensional marginal concentration, it is necessary to use the approximation $\rho_{k}\approx \phi W{\hat{\rho }}_{k}$, where ϕ represents availability and *W* is the effective width of the rectangle where the process occurs.

**Figure 2:**
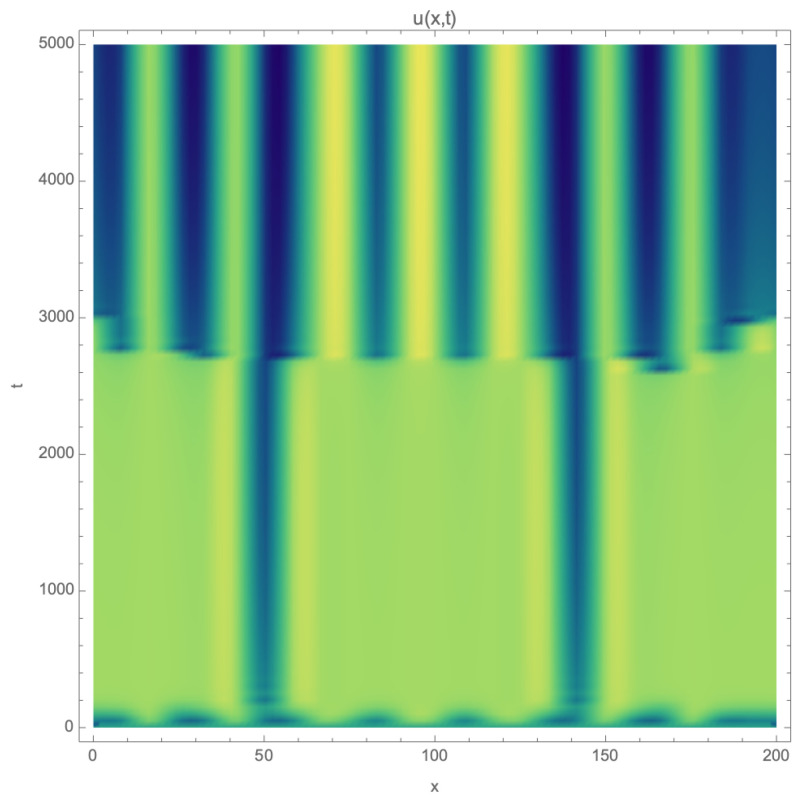
Example of spatio-temporal *U* concentration patterns in 1D reversible Gray–Scott model.

The reversible Gray–Scott system was integrated using the same parameters as in the first section, with f=0.028 and $k_{2}=0.055$, over a line of 200 points and 5000 steps, with periodic boundary conditions and an initial condition (0.5,0.25,0) perturbed by sinusoidal oscillations. Figure 2 shows patterns that appear in the one-dimensional system after some time and persist for a considerable period.

**Figure 3:**
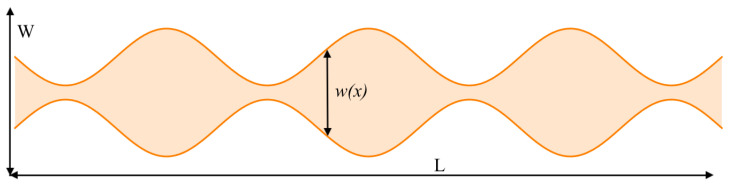
Diagram of the geometry of the constrained channel with width function $w\left(x\right)=1-0.8\sin\left(7\pi x/200\right)$, with aspect ratio 4:1, in the cell L×W.

**Figure 4:**
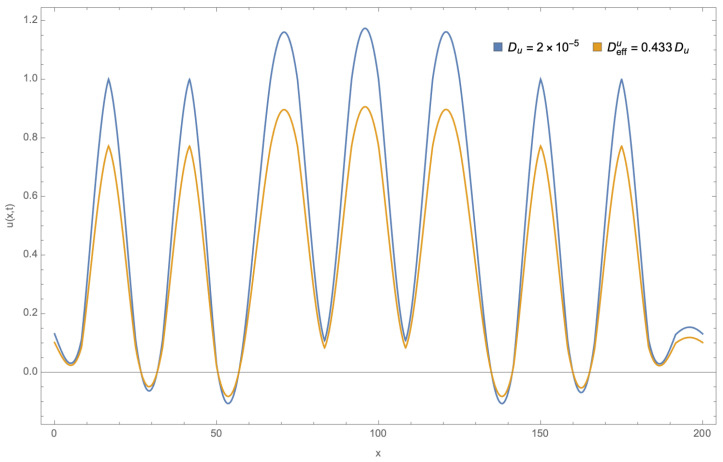
Comparison of the spatio-temporal patterns. Patterns for the u(x,y,t) concentration for both free 1D diffusion and diffusion on a constrained channel for the reversible Gray–Scott model at t=4000.

On the other hand, the system (25)–(27) was solved under the same conditions but in the constrained channel shown in Figure 3, with a width function $w\left(x\right)=1-0.8\sin\left(7\pi x/200\right)$. According to [54], this channel’s geometric parameters are (τ=1,δ=0.433,ϕ=0.386) and the corresponding effective width is W=2. It was found that the pattern’s maximum decreases due to geometric effects, as shown in Figure 4.

**Figure 5:**
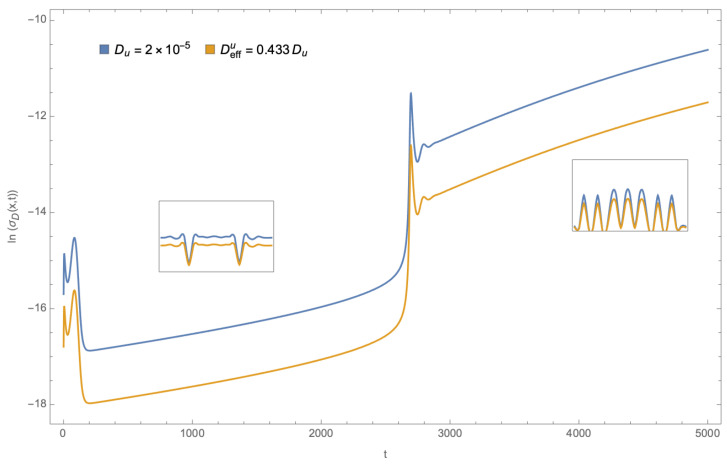
Diffusive entropy production density for free 1D diffusion, and diffusion on constrained channel at the channel’s center, as a function of time.

Finally, the density of entropy production by diffusion at the channel’s center, as a time function, is presented. Figure 5 displays both cases for the function $\ln\left(\sigma_{D}\right)$. Again, in the confined case, production is reduced compared to free diffusion. It is noted that there are two moments when this function changes abruptly, corresponding to changes in pattern formation. The monotonous part relates to partial semi-stationary patterns formed in each interval, and the characteristic pattern type is shown in the inset.

## 5. Discussion

The study of thermodynamics in the context of pattern formation within reaction–diffusion systems offers a fascinating challenge. This is particularly true when studying systems with reversible reactions, where the formation of dissipative structures can be interpreted as the coexistence and competition of two distinct states with high- and low-entropy production. Furthermore, it has been observed that confinement modifies the characteristics of the patterns in irreversible systems, depending on the geometrical parameters of the confining walls. This work examined the reversible Gray–Scott model within a narrow channel. Using the effective Fick–Jacobs model for channel diffusion, we observed that geometric modifications to the pattern are preserved in this reversible system. Additionally, we showed that the entropy production density resulting from diffusive flux is a compelling indicator of the dynamics during structure formation. Notably, while the constrained geometry of the channel influences the value of this function, its qualitative behavior remains consistent.

Our subsequent work may delve into the parameter space to examine the behavior of entropy production across various dissipative structures, similar to what was done in [20]. However, we will also address how the system’s geometry influences this behavior, through local and effective theoretical approaches. It would also be intriguing to explore the impact of geometrical effects on entropy production in non-reversible systems, as in [7,8,10], particularly on systems where the reaction kinetics are influenced by the position and distance between species, as in [49]. A comprehensive approach could involve expanding the parameter space to include the geometrical aspects of confinement, such as slope or curvature within the local Fick–Jacobs approach, as well as tortuosity, availability, or porosity, and constriction in the context of effective theory [46,54]. Such an investigation would require more demanding numerical analyses, which we aim to undertake in forthcoming studies.

## Data Availability

No new data were created or analyzed in this study. The original contributions presented in the study are included in the article.

## References

[1] Turing A.M. (1952). The chemical basis of morphogenesis. Philos. Trans. R. Soc. Lond. Ser. B.

[2] Murray J.D.L. (2003). Mathematical Biology II: Spatial Models and Biomedical Applications.

[3] Mahara H., Suematsu N.J., Yamaguchi T., Ohgane K., Nishiura Y., Shimomura M. (2004). Three-variable reversible Gray–Scott model. J. Chem. Phys..

[4] Glansdorff P., Prigogine I. (1971). Thermodynamic Theory of Structure, Stability and Fluctuations.

[5] Nicolis G., Prigogine I. (1977). Self-Organization in Non-Equilibrium System.

[6] Kondepudi D. (1998). Introduction to Modern Thermodynamics.

[7] Hanson M.P. (1974). Spatial structures in dissipative systems. J. Chem. Phys..

[8] Irvin B.R., Ross J. (1988). Calculation of the rate of entropy production for a model chemical reaction. J. Chem. Phys..

[9] Kagan E. (2010). Turing Systems, Entropy, and Kinetic Models for Self-Healing Surfaces. Entropy.

[10] López-Agudelo V.A., Barragán D. (2014). Entropy production in oscillatory processes during photosynthesis. Photochem. Photobiol. Sci..

[11] Gray P., Scott S.K. (1990). Chemical Oscillations and Instabilities, Nonlinear Chemical Kinetics.

[12] Gray P., Scott S.K. (1983). Autocatalytic reactions in the isothermal, continuous stirred tank reactor: Isolas and other forms of multistability. Chem. Eng. Sci..

[13] Escher C., Ross J. (1983). Multiranges of flow rate with bistability and limit cycles for Schlögl’s mechanism in a CSTR. J. Chem. Phys..

[14] Yoshida N. (1990). Entropy production in a chemical system involving an autocatalytic reaction in an isothermal, continuous stirred tank reactor. J. Chem. Phys..

[15] Mahara H., Susuki K., Jahan R.A., Yamaguchi T. (2008). Coexisting stable patterns in a reaction-diffusion system with reversible Gray–Scott dynamics. Phys. Rev. E.

[16] Kalantarova J., Uğrlu D. (2019). Structural stability and stabilization of solutions of the reversible three-component Gray-Scott system. Math. Meth. Appl. Sci..

[17] Mahara H., Yamaguchi T., Shimomura M. (2005). Entropy production in a two-dimensional reversible Gray-Scott system. Chaos.

[18] Mahara H., Yamaguchi T. (2010). Entropy balance in distributed reversible Gray–Scott model. Phys. D.

[19] Mahara H., Yamaguchi T. (2010). Calculation of the Entropy Balance Equation in a Non-equilibrium Reaction-Diffusion System. Entropy.

[20] Huang W.-L., Li J. (2016). Compromise between minimization and maximization of entropy production in reversible Gray–Scott model. Chem. Eng. Sci..

[21] Serna H., Muñuzuri A.P., Barragán D. (2017). Thermodynamic and morphological characterization of Turing patterns in non-isothermal reaction–diffusion systems. Phys. Chem. Chem. Phys..

[22] Silva-Dias L., Lopez-Castillo A. (2020). Turing patterns modulation by chemical gradient in isothermal and non-isothermal conditions. Phys. Chem. Chem. Phys..

[23] Mátyás L., Gaspard P. (2005). Entropy production in diffusion-reaction systems: The reactive random Lorentz gas. Phys. Rev. E.

[24] Serdyukov S. (2018). Macroscopic Entropy of Non-Equilibrium Systems and Postulates of Extended Thermodynamics: Application to Transport Phenomena and Chemical Reactions in Nanoparticles. Entropy.

[25] Berezhkovskii A., Hummer G. (2002). Single-File Transport of Water Molecules through a Carbon Nanotube. Phys. Rev. Lett..

[26] Chester A.W., Derouane E.G. (2009). Zeolite Characterization and Catalysis.

[27] Keyser U., Koeleman B., Dorp S.V., Krapf D., Smeets R., Lemay S., Dekker N., Dekker C. (2006). Direct force measurements on DNA in a solid-state nanopore. Nat. Phys..

[28] Hille B. (2001). Ion Channels of Excitable Membranes.

[29] Gouaux E., MacKinnon R. (2005). Principles of selective ion transport in channels and pumps. Science.

[30] Malgaretti P., IPagonabarraga I., Rubí J.M. (2014). Entropic Electrokinetics: Recirculation, Particle Separation, and Negative Mobility. Phys. Rev. Lett..

[31] Hänggi P., Marchesoni F. (2009). Artificial Brownian motors: Controlling transport on the nanoscale. Rev. Mod. Phys..

[32] Malgaretti P., Pagonabarraga I., Rubí J.M. (2013). Confined brownian ratchets. Front. Phys..

[33] Dagdug L., Peña J., Pompa-García I. (2024). Diffusion under Confinement. A Journey through Counterintuition.

[34] Zwanzig R. (1992). Diffusion Past an Entropy Barrier. J. Phys. Chem..

[35] Reguera D., Rubi J.M. (2001). Kinetic equations for diffusion in the presence of entropic barriers. Phys. Rev. E.

[36] Kalinay P., Percus J.K. (2005). Projection of two-dimensional diffusion in a narrow channel onto the longitudinal dimension. J. Chem. Phys..

[37] Kalinay P., Percus J.K. (2006). Corrections to the Fick-Jacobs equation. Phys. Rev. E.

[38] Bradley R.M. (2009). Diffusion in a two-dimensional channel with curved midline and varying width: Reduction to an effective one-dimensional description. Phys. Rev. E.

[39] Berezhkovskii A.M., Szabo A. (2011). Time scale separation leads to position-dependent diffusion along a slow coordinate. J. Chem. Phys..

[40] Dagdug L., Pineda I. (2012). Projection of two-dimensional diffusion in a curved midline and narrow varying width channel onto the longitudinal dimension. J. Chem. Phys..

[41] Ogawa N. (2013). Diffusion in a curved tube. Phys. Lett. A.

[42] Dagdug L., García-Chung A.A., Chacón-Acosta G. (2016). On the description of Brownian particles in confinement on a non-Cartesian coordinates basis. J. Chem. Phys..

[43] Chávez Y., Chacón-Acosta G., Dagdug L. (2018). Unbiased diffusion of Brownian particles in a helical tube. J. Chem. Phys..

[44] Chacón-Acosta G., Pineda I., Dagdug L. (2013). Diffusion in narrow channels on curved manifolds. J. Chem. Phys..

[45] Pineda I., Chacón-Acosta G., Dagdug L. (2014). Diffusion coefficients for two-dimensional narrow asymmetric channels embedded on flat and curved surfaces. Eur. Phys. J. Spec. Top..

[46] Ledesma-Durán A., León-Velasco D.A., Chacón-Acosta G., Juárez-Valencia L.H. (2023). Surface diffusion in narrow channels on curved domains. Phys. Rev. E.

[47] Ledesma-Durán A., Hernéz-Hernéz S.I., Santamaría-Holek I. (2016). Generalized Fick-Jacobs Approach for Describing Adsorption-Desorption Kinetics in Irregular Pores under Nonequilibrium Conditions. J. Phys. Chem. C.

[48] Chacón-Acosta G., Núñez-López M., Pineda I. (2020). Turing instability conditions in confined systems with an effective position-dependent diffusion-coefficient. J. Chem. Phys..

[49] Núñez-López M., Chacón-Acosta G. (2022). Pattern formation in a predator-prey system with finite interaction range in a channel-like region using the Fick-Jacobs diffusion approach. Phys. D.

[50] Chacón-Acosta G., León-Ramírez A., González-Gaxiola O. (2023). Biharmonic Fick–Jacobs diffusion in narrow channels. Phys. A.

[51] Carusela M.F., Rubi J.M. (2018). Entropy production and rectifcation efficiency in colloid transport along a pulsating channel. J. Phys. Condens. Matter.

[52] Burada P.S., Hänggi P., Marchesoni F., Schmid G., Talkner P. (2009). Diffusion in Confined Geometries. ChemPhysChem.

[53] Lifson S., Jackson J.L. (1962). On the Self-Diffusion of Ions in a Polyelectrolyte Solution. J. Chem. Phys..

[54] Ledesma-Durán A., Hernández S.I., Santamaría-Holek I. (2017). Relation between the porosity and tortuosity of a membrane formed by disconnected irregular pores and the spatial diffusion coefficient of the Fick-Jacobs model. Phys. Rev. E.

